# Fetal radiation dose of four tube voltages in abdominal CT examinations during pregnancy: A phantom study

**DOI:** 10.1002/acm2.13171

**Published:** 2021-01-15

**Authors:** Yuta Matsunaga, Tomonobu Haba, Masanao Kobayashi, Shoichi Suzuki, Yasuki Asada, Koichi Chida

**Affiliations:** ^1^ Department of Imaging Nagoya Kyoritsu Hospital Nagoya Aichi Japan; ^2^ Department of Radiological Technology Faculty of Health Sciences Tohoku University Graduate School of Medicine Sendai Miyagi Japan; ^3^ School of Health Sciences Fujita Health University Toyoake Aichi Japan

**Keywords:** fetal CT, fetal radiation dose, pregnant model, tube voltage

## Abstract

This study aimed to compare the dose and noise level of four tube voltages in abdominal computerized tomography (CT) examinations in different abdominal circumference sizes of pregnant women. Fetal radiation doses were measured with two anthropomorphic pregnant phantoms and real‐time dosimeters of photoluminescence sensors using four tube voltages for abdominal CT. The noise level was measured at the abdomen of two anthropomorphic pregnant phantoms. In the large pregnant phantom, the mean fetal doses performed using 120 and 135 kV were statistically significantly lower than the lower tube voltages (*P* < 0.05). In the small pregnant phantom, the mean fetal dose performed by 100, 120, and 135 kV was significantly lower than the lowest tube voltage tested (*P* < 0.05). The ratios of the peripheral mean dose to the centric mean dose showed that the ratios of 80 kV were the highest and those for 135 kV were the lowest in both pregnant phantoms. The ratios of the peripheral mean dose to the centric mean dose decreased as the tube voltage increased. Compared with low tube voltages, high tube voltages such as 120 and 135 kV could reduce radiation doses to the fetus without compromising the image uniformity in abdominal CT examinations during pregnancy. On low tube voltage protocols, the dose near the maternal skin surface may be increased in large pregnant women because of reduced penetration of the x rays.

## 1 INTRODUCTION

When imaging is required in the evaluation of a pregnant woman, sonography is the primary imaging technique used in medical practice worldwide. Computerized tomography (CT) is considered for pregnant women for cases in which sonographic findings are inconclusive and further imaging is deemed necessary. When CT imaging is required in the evaluation of a pregnant woman with an acute abdomen disorder such as urinary tract calculi or appendicitis, or if the prenatal diagnosis of skeletal dysplasia is suspected in a fetus, the embryo or fetus may be fully or partially in the path of the primary x‐ray beam or in its immediate vicinity. A developing fetus has a high sensitivity to ionizing radiation^1^, ^2^, ^3^; therefore, optimizing the scan parameters is crucial to achieve a diagnostically acceptable image quality at the lowest possible radiation dose.^4^, ^5^, ^6^, ^7^, ^8^, ^9^, ^10^

A previous study^11^ investigated the CT scan parameters used to diagnose fetal skeletal dysplasia in Japan and reported the various tube voltages that were being used, ranging from low to high. Although there are some reports that claim the use of a low tube voltage is helpful for radiation dose reduction in pediatric^12^, ^13^ and adult^14^, ^15^ CT, it is unclear whether the use of a low tube voltage is helpful for radiation dose reduction in fetuses. Thus, investigating the influence of the dose distribution inside the abdomen during pregnancy by varying tube voltages is important in the optimization process for the CT of pregnant women.

The abdominal circumference size of a pregnant woman changes considerably during pregnancy, which makes it important to estimate accurately the doses inside her abdomen. Many studies^16^, ^17^, ^18^, ^19^, ^20^, ^21^, ^22^ have investigated the dose evaluation for fetuses. Although some previous studies have concentrated on determining fetal doses in radiological examinations using measurements in an anthropomorphic phantom or using Monte Carlo simulations, there are no reports about concrete dose reduction methods for fetuses. Thus, the focus of this study is to compare the fetal dose under the condition of the same noise as in the fetal region.

In this study, we compared the dose and noise level of four tube voltages in abdominal CT examinations in different abdominal circumference sizes of pregnant women.

## 2 MATERIALS AND METHODS

### 2.A CT equipment and scan parameters

We used a 320‐multidetector CT (Aquilion ONE, Canon Medical Systems, Otawara, Japan) in this study. This study determined the scan parameters based on data collected from the Japanese nationwide dose survey^11^ of CT for fetal skeletal dysplasia and was performed under the following tube voltages: 80, 100, 120, and 135 kV. Measurements of each tube voltage were obtained in the x‐, y‐, and z axes with tube current modulation (TCM) (Volume EC; Canon Medical Systems). To maintain a consistent image noise level for TCM [standard deviation (SD) setting], the CT equipment determined the tube current by the scout view data. The other scan parameters are presented in Table 1. The CT equipment generated images at the following reconstruction settings for noise measurements: SD setting of 31 at a thickness of 0.5 mm; transverse slices, 5 mm; current range, 10–900 mA; and reconstruction filter, FC12. The images were reconstructed using an iterative technique, namely, the adaptive iterative dose reduction three‐dimensional (AIDR3D) technique, and the AIDR3D strength was weak.

**Table 1:** Scan parameters.

	Large pregnant phantom	Small pregnant phantom
Tube voltage (kV)	80/100/120/135	
Beam width (mm)	80 × 0.5	
Pitch factor	1.388	
Tube current (mA)	10‐900	
Rotation time (s)	0.5	
Displayed CTDI^a^_vol_ (mGy^b^) at tube voltages 80 kV/100 kV/120 kV/135 kV	3.9/3.6/3.4/3.5	3.1/2.3/2.5/2.8
Displayed DLP^c^ (mGy cm) at tube voltages 80 kV/100 kV/120 kV/135 kV	120.0/110.9/104.4/108.8	93.7/71.5/75.9/84.9

^a^ The volume computed tomography dose index (CTDI_vol_) was calculated for the 32‐cm CTDI phantom.

^b^ Milligray (mGy).

^c^ Dose‐length product (DLP).

### 2.B Pregnant model phantom and dosimeter placement

**Fig. 1:**
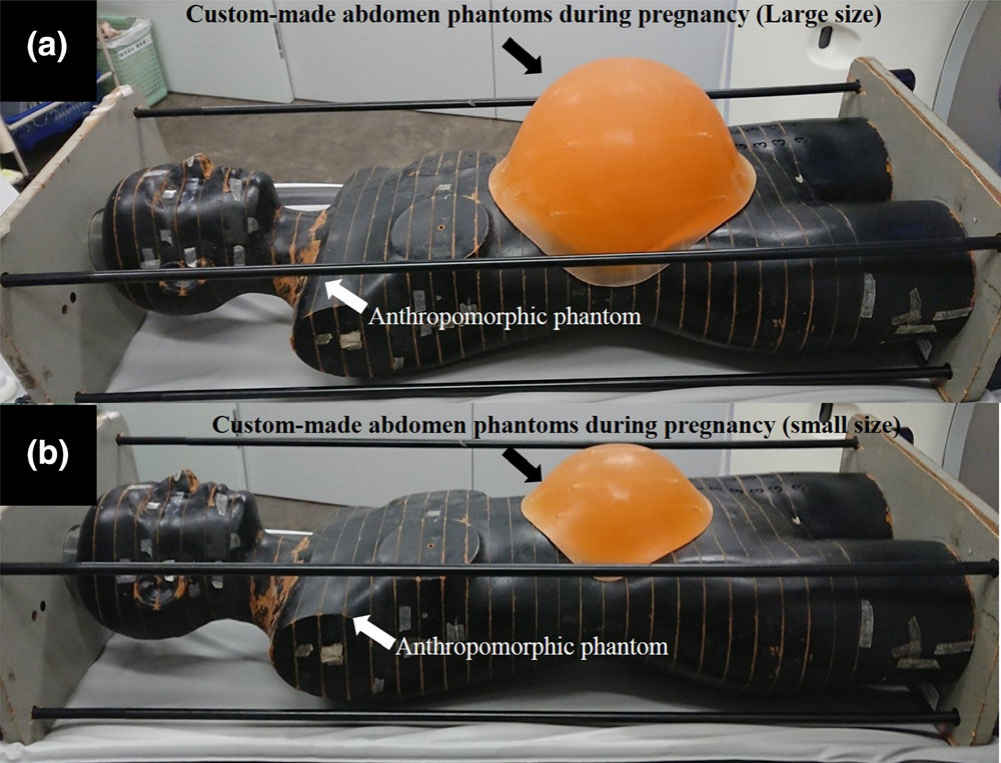
Pregnant model phantom constructed by using an anthropomorphic phantom and two different‐sized custom‐made abdomen phantoms during pregnancy used in this study. (a): large pregnant phantom; (b): small pregnant phantom.

**Fig. 2:**
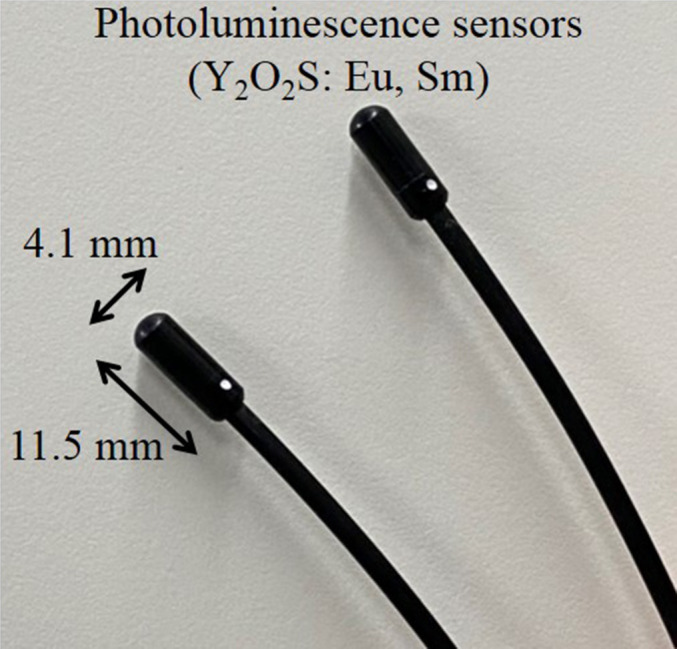
Real‐time dosimeters (RTDs) used in this study.

**Fig. 3:**
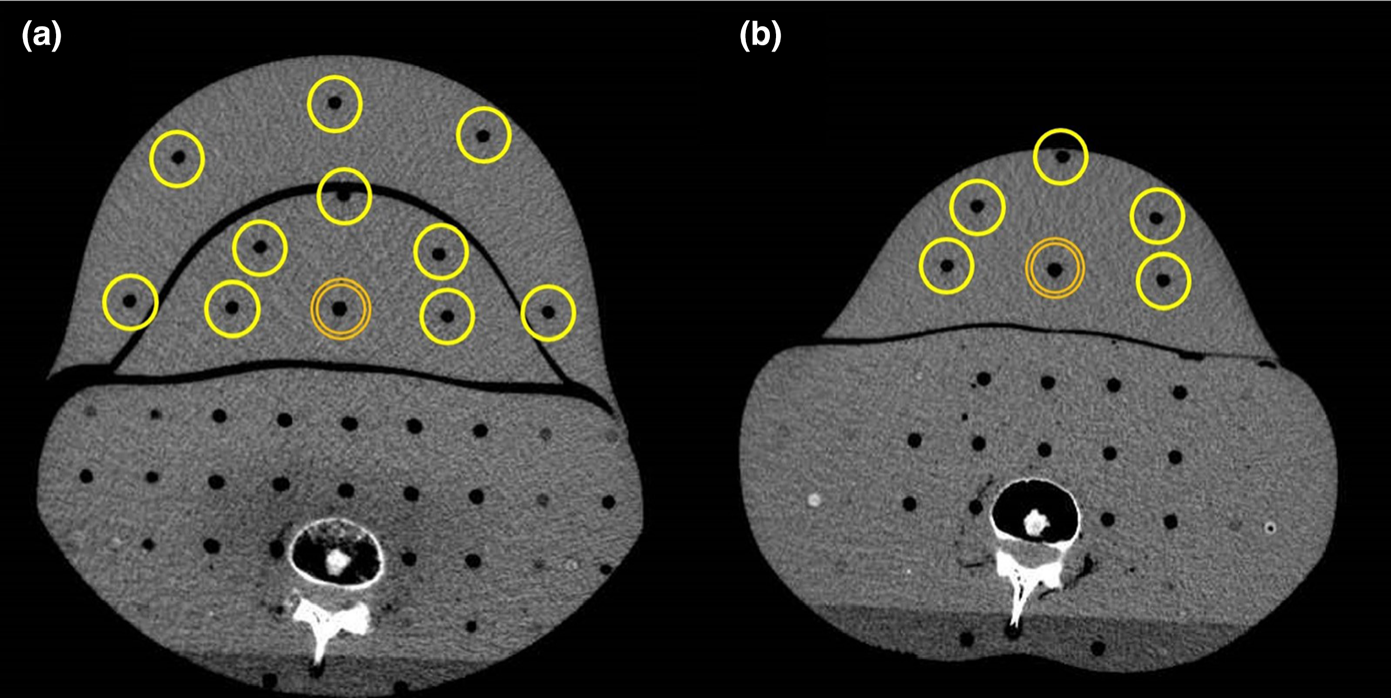
Arrangement of the real‐time dosimeters (RTDs) for measurement of the fetal radiation dose. (a): 11 measurement points at the umbilicus level of the large pregnant phantom. For the 11 measurement points on the large pregnant phantom, 1 point (a double line) was measured as the central dose and 10 points (single lines) were measured as the peripheral dose. (b): 6 measurement points at the umbilicus level of the small pregnant phantom. For the 6 measurement points on the small pregnant phantom, 1 point (a double line) was measured as the central dose and 5 points (single lines) were measured as the peripheral doses.

A pregnant model phantom was constructed using an anthropomorphic phantom (Alderson Rando phantom) and two differently sized custom‐made abdomen phantoms simulating pregnancy (Kyoto Kagaku Co. Ltd., Kyoto, Japan) (Fig. 1), hereafter referred to as the large and small pregnant phantoms. The custom‐made abdomen phantoms during pregnancy were constructed with a polyurethane resin. The specific gravity of the polyurethane resin was 1.06. The size and shape of the polyurethane resin was designed based on the abdominal size and shape collected from CT examinations of 18 pregnant patients (gestational ages of 8 to 39 weeks) in one hospital. The abdominal circumferences at the umbilicus of the large and small pregnant phantoms were 80 and 95 cm, respectively. The fetal radiation doses were measured using real‐time dosimeters (RTDs) (RD‐1000, TORECK Corporation, Kanagawa, Japan) (Fig. 2), which is a very simple and immediate reading dose measurement method without heating and annealing, compared to the conventional methods, such as thermoluminescent dosimeters^16^ and radiophotoluminescence glass dosimeters.^23^ This RTD comprised photoluminescence sensors (Y_2_O_2_S: Eu, Sm), an optical fiber cable, a photodiode, and a digital display that included the power supply, and can measure using a maximum of four sensors in one measurement.^24^, ^25^, ^26^, ^27^ The photoluminescence sensor size had a cylindrical shape with dimensions φ4.1 × 11.5 mm. RTDs were implanted at 11 and six points at the umbilical level of the large and small pregnant phantoms, respectively (Fig. 3). For the 11 measurement points on the large pregnant phantom, one point was measured as the central dose and 10 points were measured as the peripheral dose. Analogously, for the six measurement points on the small pregnant phantom, one point was measured as the central dose and five points were measured as the peripheral dose. Each measurement was performed three times to reduce random error. The scanning start angle of the x‐ray tube for each measurement was guaranteed the same by using the orbital synchronized scanning technique.

### 2.C Dose calibration and calculation

Dose calibration of RTDs for each tube voltage was performed against an ionization chamber dosimeter (9015, Radcal Corporation, Monrovia, CA, USA) with a 6‐cm^3^ thimble ionization chamber (10X5‐6, Radcal Corporation) traceable to a national standard. The chamber dosimeters and RTDs were placed adjacent to each other in the irradiated field at the same distance from the x‐ray focus. The results from the RTDs were confirmed to be equivalent to those of the ionization chamber dosimeter. The ratios of the ionization chamber dosimeter values to the RTD values at different kVs ranged from 87% to 89%. Fetal doses were calculated by multiplying the dose values obtained from the digital display by the mass energy coefficient ratio of soft tissue to air,^28^ as discussed in previous studies.^16^, ^29^

### 2.D Measurement of noise levels

**Fig. 4:**
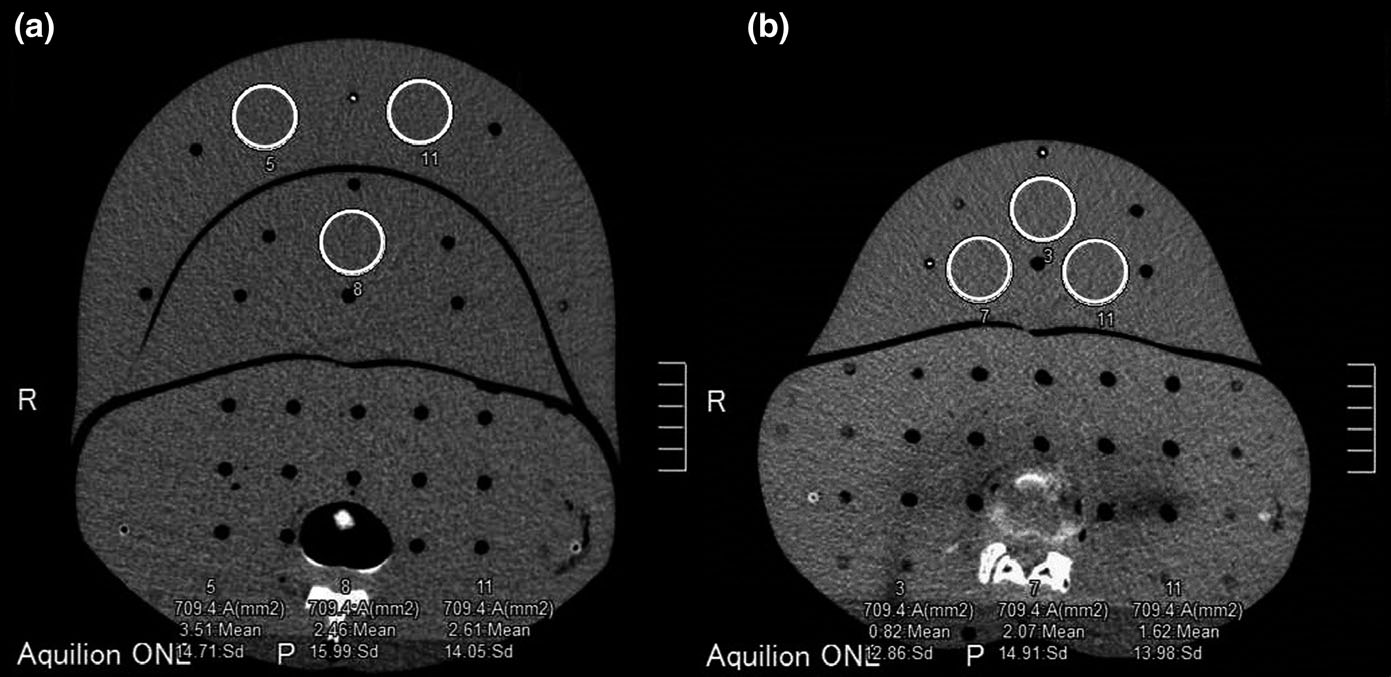
Locations of the three ROIs used to assess the image noise. (a): Large pregnant phantom. (b): Small pregnant phantom.

The image noise was measured by drawing uniform regions of interest (ROIs) in areas of the large and small pregnant phantoms in a soft tissue algorithm and expressed as the SD of Hounsfield units (HU). For each tube voltage, three 30‐mm^2^ circular ROIs were drawn at three separate slice levels and placed manually at the same visible location on the image (Fig. 4). The mean noise was calculated at each slice level. The RTD coverage spanned one image (thickness of 5 mm). “Three slices” for the noise measurement corresponded to the dosimeter’s longitudinal coverage.

### 2.E Statistics

The statistical significance of dose and image noise differences among the four tube voltages was evaluated using the Steel–Dwass procedure, which was performed using the statistical software MEPHAS (Osaka University, Osaka, Japan). A *P* < 0.05 was considered to be statistically significant.

## 3 RESULTS

### 3.A Radiation dose

**Fig. 5:**
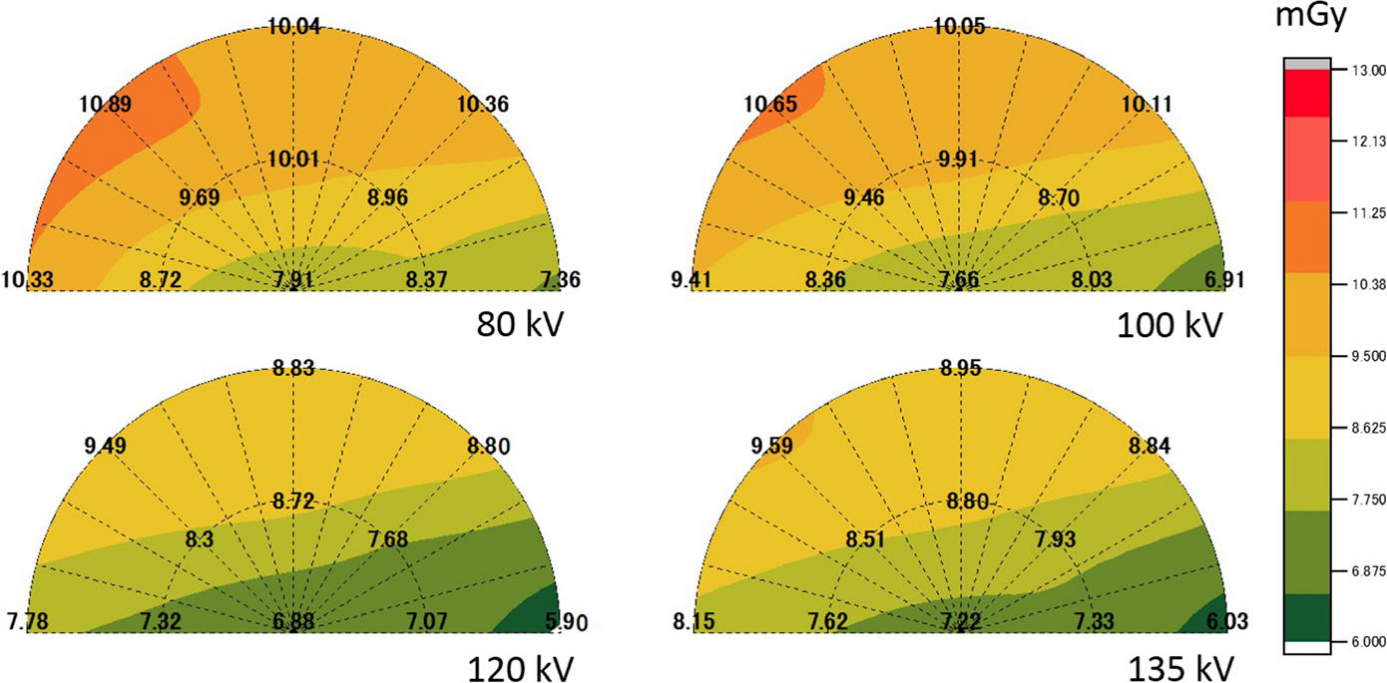
Absorbed radiation dose distributions using the four tube voltages for the large pregnant phantom.

**Fig. 6:**
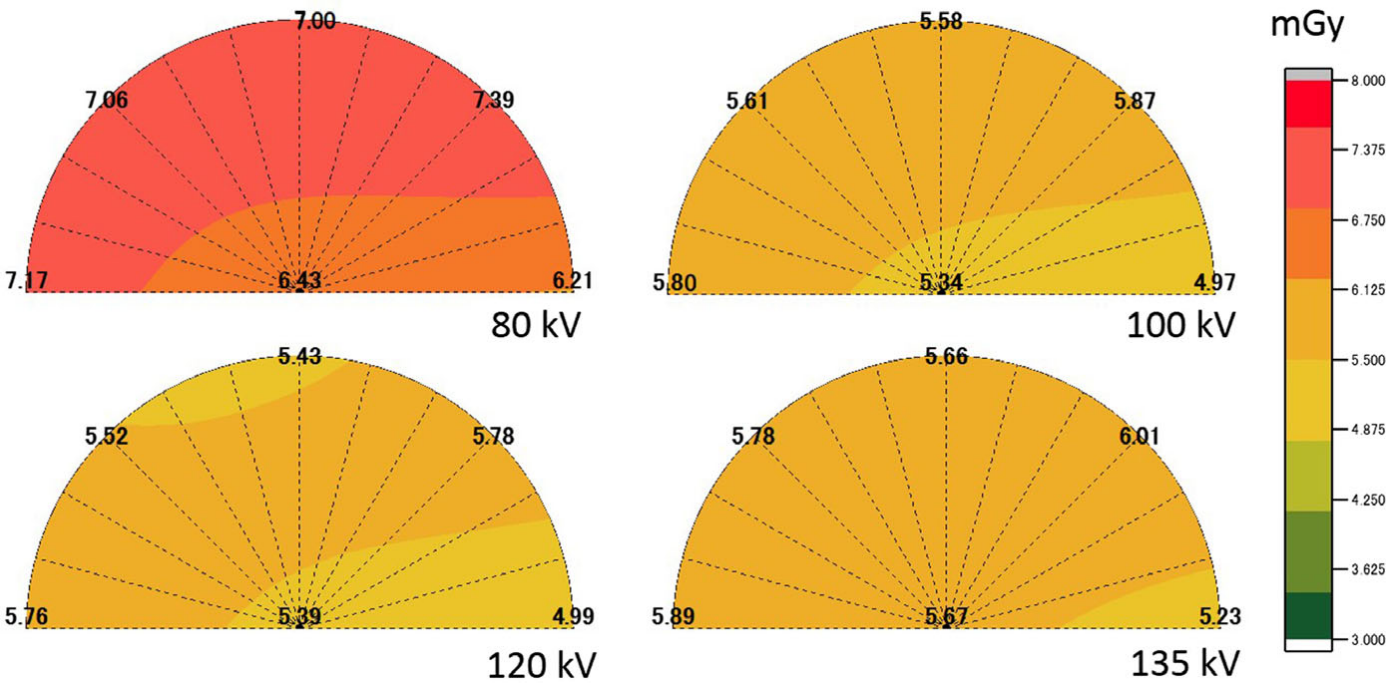
Absorbed radiation dose distributions using the four tube voltages for the small pregnant phantom.

The mean fetal radiation doses of 11 points measured using the four tube voltages for abdominal CT examinations for the large and small pregnant phantoms are shown in Table 2. In the large pregnant phantom, the mean fetal doses measured at 120 and 135 kV were statistically significantly lower than the tube voltages of 80 and 100 kV (*P* < 0.05). In the small pregnant phantom, the mean fetal doses measured at 100, 120, and 135 kV were significantly lower than the tube voltage of 80 kV (*P* < 0.05). For the mean fetal radiation doses measured using the four tube voltages, the mean fetal dose measured at 120 kV was the lowest for both pregnant phantoms. The ratios of the peripheral mean dose to the centric mean dose measured using the four tube voltages are shown in Table 3. The ratios of the peripheral mean dose to the centric mean dose showed that the ratios of 80 kV were the highest (1.18 and 1.08 times) and those for 135 kV were the lowest (1.12 and 1.01 times) in both pregnant phantoms. The ratios of the peripheral mean dose to the centric mean dose decreased as the tube voltage increased. The absorbed radiation dose distributions under the four tube voltages for the large and small pregnant phantoms are shown in Figs. 5 and 6, respectively. The low tube voltage protocols resulted in a higher peripheral dose near the maternal skin surface when compared with the condition observed under settings ≥ 120 kV for the large pregnant phantom and settings ≥ 100 kV for the small pregnant phantom.

**Table 2:** Fetal radiation doses using the four tube voltages for abdominal CT examinations during pregnancy for large and small pregnant phantoms.

Pregnant model	Tube voltage (kV)	Fetal radiation dose (mGy)		Difference to 80 kV (%)	Steel–Dwass procedure
		Mean	SD^a^		*P*‐value
Large	80	9.25	1.28	–	(80 kV, 120 kV) *P* < 0.05 (80 kV, 135 kV) *P* < 0.05 (100 kV, 120 kV) *P* < 0.05 (100 kV, 135 kV) *P* < 0.05
	100	8.96	1.30	−3.2	
	120	7.83	1.21	−15.4	
	135	8.02	1.16	−13.3	
Small	80	6.86	0.53	–	(80 kV, 100 kV) *P* < 0.05 (80 kV, 120 kV) *P* < 0.05 (80 kV, 135 kV) *P* < 0.05
	100	5.51	0.42	−19.6	
	120	5.47	0.37	−20.2	
	135	5.70	0.37	−16.9	

^a^ Standard Deviation (SD).

**Table 3:** Ratios of the peripheral mean dose to the centric mean dose measured using the four tube voltages for abdominal CT examinations during pregnancy for large and small pregnant phantoms.

Pregnant model	Tube voltage (kV)	Mean dose (mGy)		Ratio
		Centre	Peripheral	Peripheral/Centre
Large	80	7.91	9.32	1.18
	100	7.66	9.03	1.18
	120	6.88	7.88	1.14
	135	7.22	8.06	1.12
Small	80	6.43	6.94	1.08
	100	5.34	5.55	1.04
	120	5.39	5.49	1.02
	135	5.67	5.70	1.01

### 3.B Image noise

The image noise at the three points measured using the four tube voltages for the abdominal CT examinations for the large and small pregnant phantoms are shown in Table 4. There were no statistically significant differences among the four tube voltages in the SD for all ROIs in the images using the Steel–Dwass procedure.

**Table 4:** Image noise using the four tube voltages for abdominal CT examinations during pregnancy for large and small pregnant phantoms.

Pregnant model	Tube voltage (kV)	Image noise (HU^a^)		Steel–Dwass procedure
		Mean	SD^b^	*P*‐value
Large	80	16.1	1.6	*P* > 0.05
	100	14.0	1.3	
	120	14.5	1.1	
	135	14.7	1.1	
Small	80	14.7	0.7	*P* > 0.05
	100	13.9	1.0	
	120	13.8	0.8	
	135	13.7	0.8	

^a^ Hounsfield unit (HU).

^b^ Standard deviation (SD).

## 4 DISCUSSION

This study showed that the use of a high tube voltage protocol enables substantial fetal radiation dose reduction for abdominal CT examinations during pregnancy. This was accomplished while preserving image noise. Recently, there have been some reports stating that the use of low tube voltages is helpful for radiation dose reduction in pediatric^12^, ^13^ and adult^14^, ^15^ CT. However, low tube voltage protocols would be limited by patient size. A previous study^12^ reported that for patients with a body width >30 cm, dose optimized protocols required 120 kV combined with a more aggressive reduction in tube current. The abdominal circumferences of pregnant women are typically larger than those of children and general adults. In this study, the mean fetal dose using high voltages of 120 and 135 kV for the large pregnant phantom were significantly lower than those using 80 and 100 kV. On the other hand, the mean fetal dose for the small pregnant phantom was significantly lower when using ≥ 100 kV. If the abdominal circumference of a pregnant women is over 95 cm and the setting is <120 kV or the abdominal circumference is less than 80 cm and the setting is <100 kV, an increase in fetal radiation dose may result.

The increasing fetal radiation dose by low tube voltage protocols is caused by the peripheral dose near the maternal skin surface being higher than when compared with settings ≥ 120 kV for the large pregnant phantom and with settings ≥ 100 kV for the small pregnant phantom. Moreover, the ratios of the peripheral mean dose to the centric mean dose inside phantoms were high at lower tube voltages. The cause is thought to be decreased penetration in the pregnant woman as a result of x‐ray absorption near the skin surface when following low tube voltage protocols. A previous study^30^ reported that there was no correlation between fetal depth (distance from skin to fetus) and abdominal circumferences or gestational ages. There is a possibility that the position of the fetus is close to the maternal skin surface regardless of the abdominal circumferences and gestational ages. Therefore, we think that most noncontrast abdominal CT examinations during pregnancy should not use the low tube voltage protocols, but should instead use 120 or 135 kV.

A major advantage of a low tube voltage protocol is the improved image contrast by the attenuation of iodinated structures which steeply increases as the effective energy of the x‐ray spectrum approaches the iodine k‐edge (33.2 keV) with the use of a contrast medium.^12^, ^13^ For this reason, it is possible that low voltage tube protocols have a greater advantage in contrast‐enhanced CT examinations for specific indications, such as trauma, malignancy, and circulatory diseases. The most common indication for contrast CT during pregnancy is suspected pulmonary embolism. In such cases, reducing the radiation dose absorbed by the fetus and improving the image contrast are important. Thus, for contrast CT during pregnancy, using low tube voltage should be considered in accordance with the pregnant patient’s body habitus, such as the abdominal circumference.

Furthermore, the attenuation difference between low‐contrast materials is higher for low tube voltages than it is for high tube voltages. In our study, there were no statistically significant differences between the four tube voltages in the SD for all ROIs. If the images have approximately the same image noise, then low tube voltages can provide an image with a high low‐contrast resolution or contrast to noise ratio when compared with high tube voltages. However, fetal CT examinations that require the delineation of high‐contrast tissue, such as the bone, may not require low tube voltages. Radiological technicians need to select the appropriate tube voltage by setting the goal of the examinations and the position or depth of a fetus.

There is a demand for the development of standardized protocols for the optimization of CT dose.^8^, ^31^, ^32^, ^33^ As a result of recent advances in CT technology, there exist other dose reduction applications to aid pregnancy CT imaging, such as organ‐based tube current modulation (OBTCM). Through OBTCM, the exposure is lower when the x‐ray tube passes over the anterior surface of the patient. In this way, the dose to the fetus can be limited. On the other hand, this study shows that reducing the radiation dose by selecting a high tube voltage can be attained using any CT equipment in current use worldwide, without the need for new capital investment or advanced CT technology. The results of this study will be useful for the development of standardized protocols for the dose optimization of CT examinations during pregnancy.

This study was conducted only using a CT machine produced by Canon Medical Systems. The bowtie filter design thereof is significantly different from that of machines produced by other vendors; as a result, the half‐value layer is low. It is considered that, when compared with low tube voltages, high tube voltages in CT machines manufactured by other vendors could also have a certain effect on radiation doses to the fetus. However, the reduction rate in such other CT machines may be lower than that considered in this study because of the increased penetration of x rays. This study also determined the scan parameters on the basis of data collected from the Japanese nationwide dose survey^11^ of CT for fetal skeletal dysplasia. Thus, the corresponding volume CT dose indices were all below 4 mGy. The CTDIvol considered in this study is low with regard to the abdomen/pelvis studies for average patient sizes. It is unclear whether the same results will be obtained for the abdomen/pelvis studies for above‐ or below‐average patient sizes.

## 5 CONCLUSION

With low tube voltage protocols, the dose near the maternal skin surface may be increased in large pregnant women because of reduced penetration of the x rays. Compared with low tube voltages, high tube voltages such as 120 and 135 kV could reduce radiation doses to the fetus near the maternal skin surface without compromising the image uniformity in abdominal CT examinations during pregnancy. Thus, radiation dose can be reduced without any additional cost.

## CONFLICT OF INTEREST

The authors have no conflict of interest to disclose. The submitted manuscript does not contain previously published material, and is not under consideration for publication elsewhere. This study was approved by the Independent Ethics Committee of Fujita Health University.

## AUTHOR'S CONTRIBUTION

Tomonobu Haba, PhD was involved in investigation of the abdominal size and shape collected from CT examinations on pregnant patients. Masanao Kobayashi, PhD was involved in methodology of radiation dose measurements. Shoichi Suzuki, PhD was involved in methodology of radiation dose measurements. Yasuki Asada, PhD was involved in methodology of radiation dose measurements. Koichi Chida, PhD was involved in methodology, review, and editing.
